# Baseline Neurocognitive Performance and Symptoms in Those With Attention Deficit Hyperactivity Disorders and History of Concussion With Previous Loss of Consciousness

**DOI:** 10.3389/fneur.2019.00396

**Published:** 2019-04-24

**Authors:** Sarah Kaye, Mark H. Sundman, Eric E. Hall, Ethan Williams, Kirtida Patel, Caroline J. Ketcham

**Affiliations:** ^1^Mailman Research Center, McLean Hospital, Belmont, MA, United States; ^2^Department of Psychology, University of Arizona, Tucson, AZ, United States; ^3^Department of Exercise Science, Elon University, Elon, NC, United States; ^4^Office of the Dean of Students, Elon University, Elon, NC, United States; ^5^Department of Sports Medicine, Elon University, Elon, NC, United States

**Keywords:** mild traumatic brain injury, concussion management, pre-existing conditions, ADHD, LOC

## Abstract

Previous consensus statements on sports concussion have highlighted the importance of Attention Deficit Hyperactivity Disorder (ADHD) and loss of consciousness (LOC) as risk factors related to concussion management. The present study investigated how self-reported history of either ADHD diagnosis or history of previous concussion resulting in LOC influence baseline neurocognitive performance and self-reported symptoms. This analysis was performed retrospectively on data collected primarily from student-athletes, both Division 1 and club sports athletes. The dataset (*n* = 1460) is comprised of college students (age = 19.1 ± 1.4 years). Significant differences were found for composite scores on the ImPACT for both history of concussion (*p* = 0.016) and ADHD (*p* = 0.014). For concussion history, those with a previous concussion, non-LOC, performed better on the visual motor speed (*p* = 0.004). Those with diagnosis of ADHD performed worse on verbal memory (*p* = 0.001) and visual motor speed (*p* = 0.033). For total symptoms, concussion history (*p* < 0.001) and ADHD (*p* = 0.001) had an influence on total symptoms. Those with ADHD reported more symptoms for concussion history; those with previous LOC concussion reported more symptoms than those with non-LOC concussion (*p* = 0.003) and no history (*p* < 0.001). These results highlight the importance of baseline measures of neurocognitive function and symptoms in concussion management in order to account for pre-existing conditions such as ADHD and LOC from previous concussion that could influence these measures.

## Introduction

In recent years, concussion research has quickly made its way to the forefront of the medias' attention and has been deemed a public health issue (1) and a silent, global epidemic (2). Each year it is estimated that there are between 1.6 and 3.8 million sports-related concussions (3) and they account for ~5–9% of all sports-related injuries (4, 5). The use of neurocognitive measures has previously been deemed a cornerstone of concussion management (6, 7). In a recent consensus statement, the implementation of baseline measures of neurocognitive performance was believed to be helpful and useful for concussion management, but not mandatory (8).

Among the factors that have been hypothesized to influence recovery from concussion and may warrant additional research include diagnosis of Attention Deficit Hyperactivity Disorder (ADHD) and having a previous concussion that resulted in loss of consciousness (LOC) (6, 7). Recent studies have found that previous diagnosis of ADHD influences neurocognitive performance (9–13) and total symptom scores at baseline assessment (9–12). The influence of concussion history with LOC on recovery to subsequent concussions has been examined less than ADHD despite concussions at one time being graded with LOC being a main component of concussion diagnosis (14). Currently, however, it is estimated that LOC only occurs in 5–10% of sports-related concussions (7, 15, 16). Though concussions are no longer classified solely on the presence of LOC, evidence indicates that LOC is a potential modifier of concussion recovery (7, 17, 18). However, only one known study has examined whether LOC influences performance on neurocognitive measures (19). That study examined neurocognitive performance following a concussion and did not find any significant differences between groups (concussions with LOC and without LOC). This previous research suggests that those who are involved in concussion management should take into consideration factors such as ADHD and previous history of LOC when treating patients (7, 8, 20).

The current study examined the influence of self-reported diagnosis of ADHD and history of LOC at the time of concussion on neurocognitive performance and reporting of symptoms at a baseline pre-season assessment.

## Materials and Methods

### Participants

Data was initially collected from 1514 college students, but 54 participants were taken out because of having an invalid baseline. The final sample include 1460 college students (665 females, 795 males; mean age = 19.1 ± 1.4 years). Most were student-athletes (Division I varsity = 565, club, *n* = 821) as well as dance majors (*n* = 33) who were tested as a result of university's concussion management protocol, plus an additional 41 students who were tested as controls for other projects. All participants were 18 years of age or older. The study received Institutional Review Board approval before testing began and all participants completed informed consent forms to participate in this study.

### Measures

ImPACT (version 2.1) is a commonly used tool for concussion management which assesses neurocognitive performance and symptoms (21).

The self-reported demographic data was used to determine history of ADHD diagnosis, number of total concussions, and number of concussions with LOC. ImPACT contains six modules to measure neurocognitive performance; these modules include: word discrimination (measures attention and verbal memory), design memory (measures attention and visual memory), X's and O's (measures working memory, visual memory and processing speed), symbol matching (measures visual processing speed, learning and memory), color match (measures choice reaction time, impulse control, and inhibition), and three letter memory (measures working memory and visual motor response speed). From these six modules, ImPACT calculates four composite scores: verbal memory, visual memory, visual motor speed, and reaction time. Total symptom score was based on responses to the 22 symptom list which uses a 7-point likert scale (ranging from 0 to 6 on severity); therefore, the symptom score that is reported is based on number and severity of symptoms Previous research has suggested that the scores from the ImPACT may have somewhat poor reliability (21), but most studies have demonstrated good test-retest reliability for this measure (13, 15).

### Procedures

The study was performed with a retrospective analysis of baseline pre-season ImPACT tests from spring 2011 until spring 2016. The Division I student-athletes, club athletes, and dancers all completed the ImPACT assessment as part of the university's concussion management protocols, which are required for athletes to complete prior to participation in their respective athletic endeavor. The “other” group were college students that participated in previous research as control subjects and had completed the same baseline ImPACT assessment. For most of the Division I student-athletes, the ImPACT was administered in a quiet computer laboratory with no more than two student-athletes at a time. For the rest of the sample the ImPACT was administered in a larger computer lab and often in a group setting. Testing was administered at various times during the day depending on availability of the participants. Generally, the ImPACT was completed in 20–30 min.

### Statistical Analysis

All data were analyzed using SPSS 23 (IBM) with an alpha level of <0.05. A Multivariate Analyses of Variance (MANOVA) was conducted with self-reported previous diagnosis of ADHD and history of a concussion (no history, history with LOC, history without LOC) as independent variables, with dependent variables derived from the ImPACT composite scores (e.g., verbal memory, visual memory, visual motor speed, and reaction time). Similarly, an ANOVA was conducted with self-reported previous diagnosis of ADHD and a history of concussion (no history, history with LOC, history without LOC) as independent variables, and total symptoms score derived from the ImPACT assessment as the dependent variable. LSD *post-hoc* tests were performed to determine where significant differences occurred.

## Results

Of the 1,460 participants, 168 (11.5%) self-reported a previous diagnosis of ADHD and 408 (27.9%) of the participants self-reported having a previous concussion. For the ADHD group, 48.2% (81 of 168) self-reported taking at least one medication typically prescribed for treatment of ADHD. Of the 408 participants with a concussion, 112 (27.4% of those with concussion and 7.7% of total sample) reported having history of concussion with LOC.

The MANOVA for the ImPACT composite scores found a statistically significant main effect for those diagnosed with ADHD [*F*_(4, 1451)_ = 3.16, *Wilks'* λ = 0.99, *p* = 0.014] as well as for concussion history [*F*_(8, 2902)_ = 2.34, *Wilks'* λ = 0.99, *p* = 0.016], but not a significant interaction for concussion history and ADHD (see Table 1). For those with a previous diagnosis of ADHD, there was a significant difference for performance on verbal memory [*F*_(1, 1454)_ = 10.41, *p* = 0.001] and visual motor speed [*F*_(1, 1454)_ = 4.54, *p* = 0.033] as those who reported a previous diagnosis of ADHD performed worse on those measures.

**Table 1:** Means (±SE) for ImPACT composite scores and total symptoms for concussion history and ADHD.

**Group**	**Verbal memory**	**Visual memory**	**Visual motor speed**	**Reaction time**	**Total symptoms**
No concussion	84.5 ± 0.5	73.6 ± 0.7	40.3 ± 0.3^a^	0.583 ± 0.004	4.7 ± 0.4^a^
Concussed without LOC	86.1 ± 0.8	76.0 ± 1.1	42.3 ± 0.5^a,b^	0.568 ± 0.006	5.7 ± 0.6^b^
Concussed with LOC	82.2 ± 1.5	75.3 ± 1.9	40.0 ± 0.9^b^	0.589 ± 0.011	9.4 ± 1.1^a,b^
No ADHD	86.2 ± 0.4^a^	75.8 ± 0.5	41.6 ± 0.3^a^	0.574 ± 0.003	5.1 ± 0.3^a^
ADHD	82.4 ± 1.1^a^	74.2 ± 1.4	40.1 ± 0.7^a^	0.587 ± 0.008	8.1 ± 0.8^a^

*Those with similar letters designate statistically significant differences between groups, p < 0.05*.

For concussion history, univariate analyses found significant differences for and visual motor speed [*F*_(2, 1454)_ = 5.52, *p* = 0.004], but not for the other scales. Those with history of non-LOC concussion performed better on visual motor speed than both those who reported history of concussion with LOC (*p* = 0.023) and those with no history of concussion (*p* = 0.001). While not significant, there were some trends for verbal memory [*F*_(2, 1454)_ = 2.84, *p* = 0.059] and reaction time [*F*_(2, 1454)_ = 2.39, *p* = 0.092] related to concussion history.

An ANOVA for total symptoms score revealed significant main effects for ADHD [*F*_(1, 1454)_ = 11.72, *p* = 0.001] and LOC [*F*_(2, 1454)_ = 8.69, *p* < 0.001], but not a significant interaction between the two (see Table 1). Those with a previous diagnosis of ADHD reported more symptoms than those without ADHD. Total symptom scores were higher for those with a history of concussion resulting in LOC than those with a previous concussion absent of LOC (*p* = 0.003) as well as those with no history of any concussion (*p* < 0.001).

## Discussion

Previous research has explored the extent to which ADHD influences neurocognitive performance and the self-reporting of symptoms at baseline (9–12), but there has been relatively little investigation into how history of a concussion with LOC influences these measures (19). This research study sought to further the research that has been previously performed to better understand how history of ADHD and LOC may influence baseline measures of neurocognitive performance and self-reported symptoms when student-athletes are assessed prior their participation in collegiate sports. This enables the examination of the potential residual effects of LOC and ADHD history on neurocognitive and symptom measures at baseline, which may ultimately influence concussion management strategies.

Our sample had 11.5% of our population report a previous diagnosis with ADHD, which is higher than the 4.2–8.1% reported in a recent review of literature on ADHD in athletes (20). While our percentage is higher than that with athletes, it is more consistent with previous research which shows the percentage to be around 11% in adolescents (22, 23). This could be due to the inclusion of non-varsity athletes (e.g., club athletes and dancers). In agreement with previous research, those with ADHD also reported significantly higher symptoms at baseline than participants without ADHD (9–11). The symptoms for ADHD closely resemble those that are commonly associated acutely with concussions, which may make the two difficult to distinguish. One possible explanation for this finding is a self-report bias as the population of participants with ADHD may be more accustomed to neuropsychological exams and reporting symptoms in their interactions with medical professionals. The neurocognitive test results showed that significant differences between those with ADHD and those without ADHD were that those without ADHD had significantly higher verbal memory and visual motor scores. This finding is congruent with other previous research (9–13, 24). However, it is important to note that these findings are based on self-reported diagnosis of ADHD and was not verified by a physician and could have possibly influenced our results.

Results from the neurocognitive assessments demonstrate that those reporting a history of concussions without LOC had significantly higher visual motor scores than both the LOC and no history of concussion group. However, concussion symptoms were significantly higher for those who had been concussed with LOC compared to the other two groups. These findings for the influence of LOC on neurocognitive performance is opposite of previous work which has found history of LOC to not influence neuropsychological performance (19, 25).

These results suggest that LOC at time of concussion may lead to residual deficits and symptoms, and, further, that chronic effects from previous concussions with LOC should be investigated further. This is consistent with current recommendations that LOC should be considered in concussion management (7, 8). One future direction could be investigating which, if any, specific brain structures and/or networks are involved in LOC to help elucidate the influence of LOC on neurocognitive performance and symptom reporting (26).

This research also raises a question about the combination of ADHD and a history of concussions that resulted in LOC. ADHD is known for having an influence on axonal integrity, white matter and function of certain areas of the brain (27–29), so it is possible that people with ADHD are more likely to experience concussions (30) or those with history of concussions to also report symptoms of ADHD (31). It might also be that these alterations in function and structure of the brain could predispose them to pathophysiology associated with LOC (26).

In conclusion, this research suggests that ADHD and LOC should be considered in concussion management as both of these pathologies may influence measures from baseline concussion testing, symptoms and neurocognitive performance, that are commonly used in concussion management.

## Ethics Statement

Elon University IRB 13-017; 17-008; 17-043. This study was carried out in accordance with the recommendations of name of guidelines, name of committee with written informed consent from all subjects. All subjects gave written informed consent in accordance with the Declaration of Helsinki. The protocol was approved by the name of committee.

## Author Contributions

SK, EH, and CK were involved in development of research idea. SK, MS, EH, and CK were involved in writing of the manuscript. All authors reviewed the manuscript and assisted in data collection.

### Conflict of Interest Statement

The authors declare that the research was conducted in the absence of any commercial or financial relationships that could be construed as a potential conflict of interest.
